# **­­­**Proteomic analysis of H3K36me3 and PSIP1/p75 (LEDGF) complexes reveal their wider role in DNA repair

**DOI:** 10.12688/wellcomeopenres.11589.1

**Published:** 2017-09-12

**Authors:** Madapura M. Pradeepa, Gillian C.A. Taylor, Alex von Kriegsheim

**Affiliations:** 1 MRC Human Genetics Unit, MRC Institute of Genetics and Molecular Medicine, University of Edinburgh, Edinburgh, EH4 2XU, UK; 2 School of Biological Sciences, University of Essex, Colchester, CO4 3SQ, UK

**Keywords:** H3K36me3, PSIP1, LEDGF, SILAC, mass spectrometry, DNA repair, MOF

## Abstract

**Background:** Trimethylation at histone H3 at lysine 36 (H3K36me3) is associated with expressed gene bodies and recruit proteins implicated in transcription, splicing and DNA repair. PC4 and SF2 interacting protein ( *PSIP1/LEDGF*) is a transcriptional coactivator, possesses a  H3K36me3 reader PWWP domain. Alternatively spliced isoforms of PSIP1 binds to H3K36me3 and suggested to function as adaptor proteins to recruit transcriptional modulators, splicing factors and proteins that promote homology directed repair (HDR), to H3K36me3 chromatin.

**Methods: **We performed chromatin immunoprecipitation of H3K36me3 followed by quantitative mass spectrometry to identify proteins associated with H3K36 trimethylated chromatin in mouse embryonic stem cells (mESCs). Furthermore, we performed stable isotope labelling with amino acids in cell culture (SILAC) for a longer isoform of PSIP1 (p75) and MOF/KAT8 in mESCs and mouse embryonic fibroblasts (MEFS).

**Results:** Proteomic analysis of H3K36me3 chromatin show association of proteins involved in transcriptional elongation, RNA processing and DNA repair with H3K36me3 chromatin. Furthermore, we show DNA repair proteins like PARP1, gamma H2A.X, XRCC1, DNA ligase 3, SPT16, Topoisomerases and BAZ1B are predominant interacting partners of PSIP1/p75. We validated the association of PSIP1/p75 with gamma H2A.X, an early marker of DNA damage and also demonstrated accumulation of damaged DNA in PSIP1 knockout MEFs.

**Conclusions:** In contrast to the previously demonstrated role of H3K36me3 and PSIP1/p75 in promoting HDR in mammals, our data supports the wider role of H3K36me3 and PSIP1 in maintaining the genome integrity by recruiting several DNA repair proteins to transcribed gene bodies.

## Introduction

**Figure 1.**
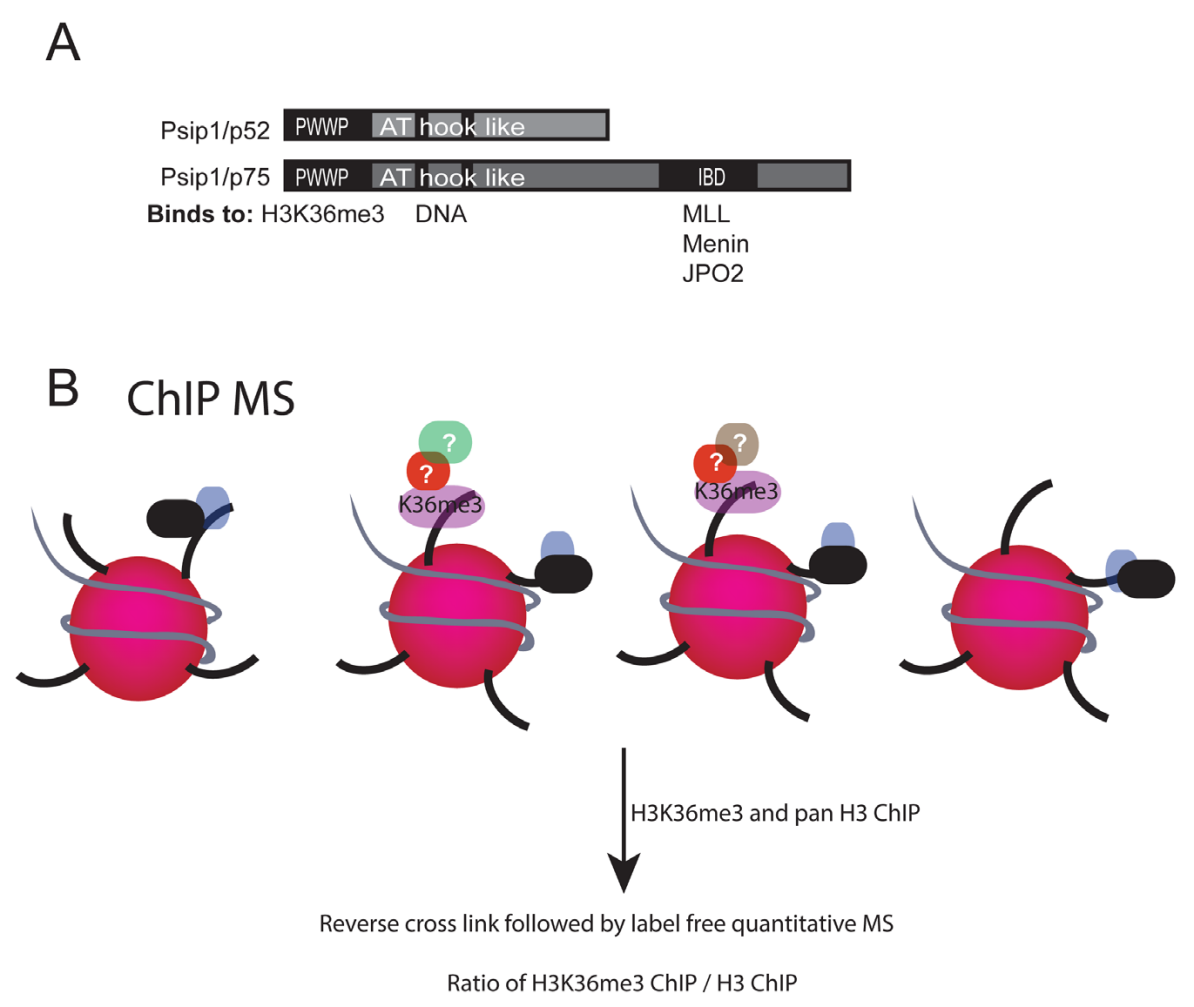
PSIP1/p75 domains and its functional interactors. ( **A**) Cartoon of PSIP1 p52 and p75 isoforms showing methylated histone binding PWWP domain, DNA binding AT hook domain at the N-terminus and also the integrase binding domain (IBD) domain at the C-terminus of p75 that interacts with MLL1, Menin and JPO2. ( **B**) Illustration showing crosslinked chromatin immunoprecipitation (xChIP) followed by mass spectrometry (MS) to identify proteins associated with histone modifications.

PC4 and SF2 interacting protein ( *PSIP1*) encode two splice variants – a shorter isoform called p52 and a longer isoform called p75. The N-terminal PWWP and adenine-thymine (AT) hook-like DNA binding domains are common to both isoforms ( Figure 1A). The PWWP domain binds specifically to Histone H3 trimethylated at lysine 36 (H3K36me3); both PSIP1 isoforms and H3K36me3 co-occur at expressed gene bodies ( Pradeepa *et al.*, 2012; Pradeepa *et al.*, 2014; van Nuland *et al.*, 2013a). The C-terminal integrase binding domain is unique to p75, and is shown to interact with HIV integrase ( Cherepanov *et al.*, 2003; Ciuffi *et al.*, 2005); the same domain binds to the mixed lineage leukaemia proteins (MLL1, MLL2 and Menin) ( Pradeepa *et al.*, 2014; van Nuland *et al.*, 2013b; Yokoyama & Cleary, 2008). Psip1/p75 is a multifunctional chromatin protein that has been implicated in regulation of homeotic genes, cell survival, cancers and autoimmune diseases, and has also been shown to promote homology derived repair (HDR) by recruiting C-terminal binding protein interacting protein (CtIP) to double stranded breaks (DSB) ( Basu *et al.*, 2012; Ciuffi *et al.*, 2005; Daniels *et al.*, 2005; Desfarges & Ciuffi, 2010; Pradeepa *et al.*, 2014; Sutherland *et al.*, 2006).

We have previously demonstrated the role of the p52 isoform in modulating alternative splicing by recruiting splicing factors to H3K36 trimethylated chromatin through its N terminal PWWP domain ( Pradeepa *et al.*, 2012). Work from other groups has shown that the PSIP1/p75 isoform interacts with: JPO2 (also known as R1, RAM2 and CDCA7L) ( Bartholomeeusen *et al.*, 2007), pogo transposable element with zinc finger domain (PogZ) ( Bartholomeeusen *et al.*, 2009), the activator of the S-phase kinase complex (CDC7-ASK) ( Hughes *et al.*, 2010), methyl CpG binding protein 2 (MeCP2) ( Bartholomeeusen *et al.*, 2009), and CtIP ( Daugaard *et al.*, 2012) and interacts-with-Spt6 (IWS1) ( Tesina *et al.*, 2015), in different genomic location or cellular contexts.

Several pieces of evidence support the role of H3K36me3 in the DNA damage response. In mammals, SETD2 mediated H3K36me3 is shown to recruit PSIP1/p75 to express genes, which upon DNA damage recruits the repair factors CtIP and RAD51 to facilitate HDR ( Aymard *et al.*, 2014; Daugaard *et al.*, 2012; Pfister *et al.*, 2014). All this evidence supports a model in which PSIP1 is constitutively anchored to H3K36me3 chromatin at expressed gene bodies through its PWWP domain. Upon DNA damage, chromatin bound PSIP1/p75 recruits CtIP and RAD51, which promotes HR repair by efficient resection, and protects these vulnerable regions of the genome from DNA damage. In the absence of SETD2 or H3K36me3, the chromatin association of PSIP1 is reduced, and DNA damage induced recruitment of repair proteins is impaired, leading to reduced resection and HDR. Another H3K36me3 reader – MRG15 – has been shown to recruit the partner and localiser of BRCA2 (PALB2) complex to undamaged chromatin ( Bleuyard *et al.*, 2017). Constitutive association of PALB2 to H3K36me3 chromatin at expressed gene bodies facilitates immediate availability of PALB2 upon DNA damage during active transcription and DNA replication. H3K36me3 is also shown to promote DNA mismatch repair by recruiting the mismatch recognition protein MutSα through its PWWP domain ( Li *et al.*, 2013). In contrast to mammalian studies, in budding and fission yeasts, H3K36me3 promotes non homologous end joining (NHEJ) and inhibits HDR (reviewed in ( Jha *et al.*, 2014). Similar to yeast studies, H3K36me2, catalysed by SETMAR/Metnase also promotes NHEJ in human cells ( Fnu *et al.*, 2011). This suggests a complex role of H3K36 methylation in DNA repair choice and genome stability. SETD2, the only enzyme responsible for H3K36 trimethylation is mutated in cancers and is proposed to function as tumor suppressor ( Li *et al.*, 2016; Zhu *et al.*, 2014). The H3K36me3 reader – PSIP1 – is implicated in a variety of cancers ( Basu *et al.*, 2016; Daniels *et al.*, 2005; French *et al.*, 2016; Yokoyama & Cleary, 2008) and also implicated in resistance to chemotherapy induced cell death in prostate cancer ( Mediavilla-Varela *et al.*, 2009). This suggests that H3K36me3 and PSIP1 play an important role in DNA repair and that dysregulation of this pathway could cause human cancer.

We hypothesised that PSIP1 isoforms function as an adaptor protein to recruit various proteins involved in transcription, splicing and DNA repair to H3K36me3 sites via PWWP domain. We have previously shown that the p52 isoform binds to H3K36me3 and recruits splicing factors to exons. Here, we performed formaldehyde crosslinked chromatin immunoprecipitation (ChIP) followed by label-free quantitative mass spectrometry (xChIP-qMS) to identify proteins associated with H3K36me3 chromatin. We find several proteins implicated in transcriptional elongation, RNA processing and DNA repair associated with H3K36me3 *in vivo*. Furthermore, SILAC proteomics analysis of endogenous PSIP1/p75 complex shows that several DNA repair proteins interact with p75. We also detect a higher level of DNA damage in mouse embryonic fibroblasts (MEFs) derived from a *Psip1* knockout mouse ( *Psip1 ^–/–^*). We propose a wider role of H3K36me3 and PSIP/p75 in maintaining genome integrity at the site of transcription.

## Results

### xChIP-qMS identifies H3K36me3 associated proteins *in vivo*

H3K36me3 is associated with actively transcribed gene bodies, preferentially exons of the expressed genes, suggesting its role in splicing and transcriptional elongation. H3K36me3 peptide pulldown followed by SILAC-MS identified that the PWWP domain has a putative H3K36me3 reader domain ( Vermeulen *et al.*, 2010). In order to capture the proteins that transiently and stably associate with H3K36me3, formaldehyde cross linked mouse embryonic stem cells (mESCs) were treated with hypotonic buffer to prepare nuclei and sonicated to obtain soluble chromatin, and were immuno-precipitated using H3K36me3 and pan H3 antibodies bound to magnetic beads ( Figure 1B).

**Figure 2.**
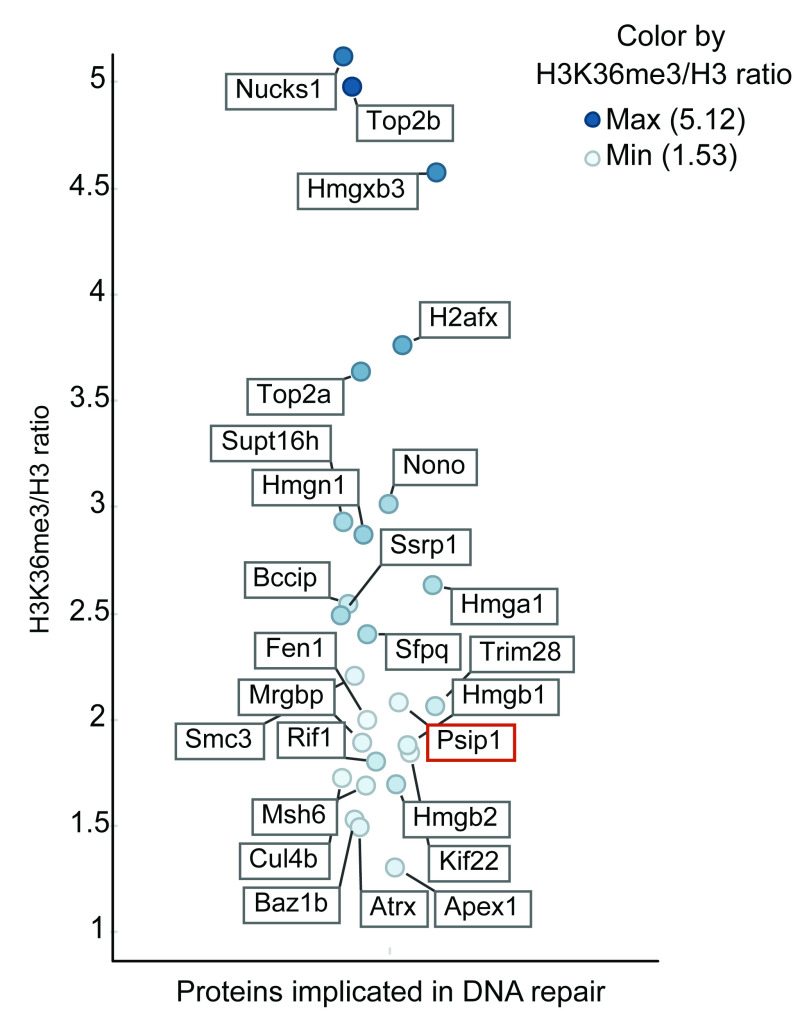
H3K36me3 associated proteins that are implicated in DNA repair. The label-free mass spectrometry quantitative output values assigned to each protein following immunoprecipitation from the mouse embryonic stem cells. The list of proteins associated with DNA repair function with the H3K36me3 vs H3 ratio of more than 1.5 (y-axis) are plotted (full list of proteins in Dataset 1). Horizontal scatter was added only to aid visibility of each protein and has no data correlate. The position of PSIP1 protein is highlighted in red.

Label-free quantitative mass spectrometry analysis of two replicate ChIPs resulted in identification of several proteins, implicated in replication, transcription, RNA processing and DNA repair, which were quantitatively enriched in ChIP with anti-H3K36me3 normalised to anti-pan H3 antibodies ( Figure 2 and Dataset 1). Association of RNA processing proteins with H3K36me3 is consistent with the role of PSIP1/p52 in binding to H3K36me3 and recruiting several RNA processing proteins to exons of expressed genes to modulate alternative splicing ( Pradeepa *et al.*, 2012). Similarly, MRG15 binds to H3K36me3 and recruits PTBP to modulate alternative splicing ( Luco *et al.*, 2010) and another PWWP domain protein BS69 binds to H3K36me3 and recruits splicing factors to influence splicing pattern ( Guo *et al.*, 2014). Since H3K36me3 is located at expressed gene bodies, it is not surprising that we find several proteins implicated in transcription and transcriptional elongation ( Dataset 1). Interestingly, we found 26 proteins that are implicated in DNA repair and are associated with H3K36me3 *in vivo*, with >1.5 ratio of H3K36me3 ChIP/ H3 ChIP ( Figure 2 and Dataset 1). These include known interactors of H3K36me3 – PSIP1, SPT16, SSRP1 and MSH6 ( Carvalho *et al.*, 2013; Li *et al.*, 2013; Pradeepa *et al.*, 2012; Pradeepa *et al.*, 2014).

### SILAC proteomics of PSIP1/p75 complex

**Figure 3.**
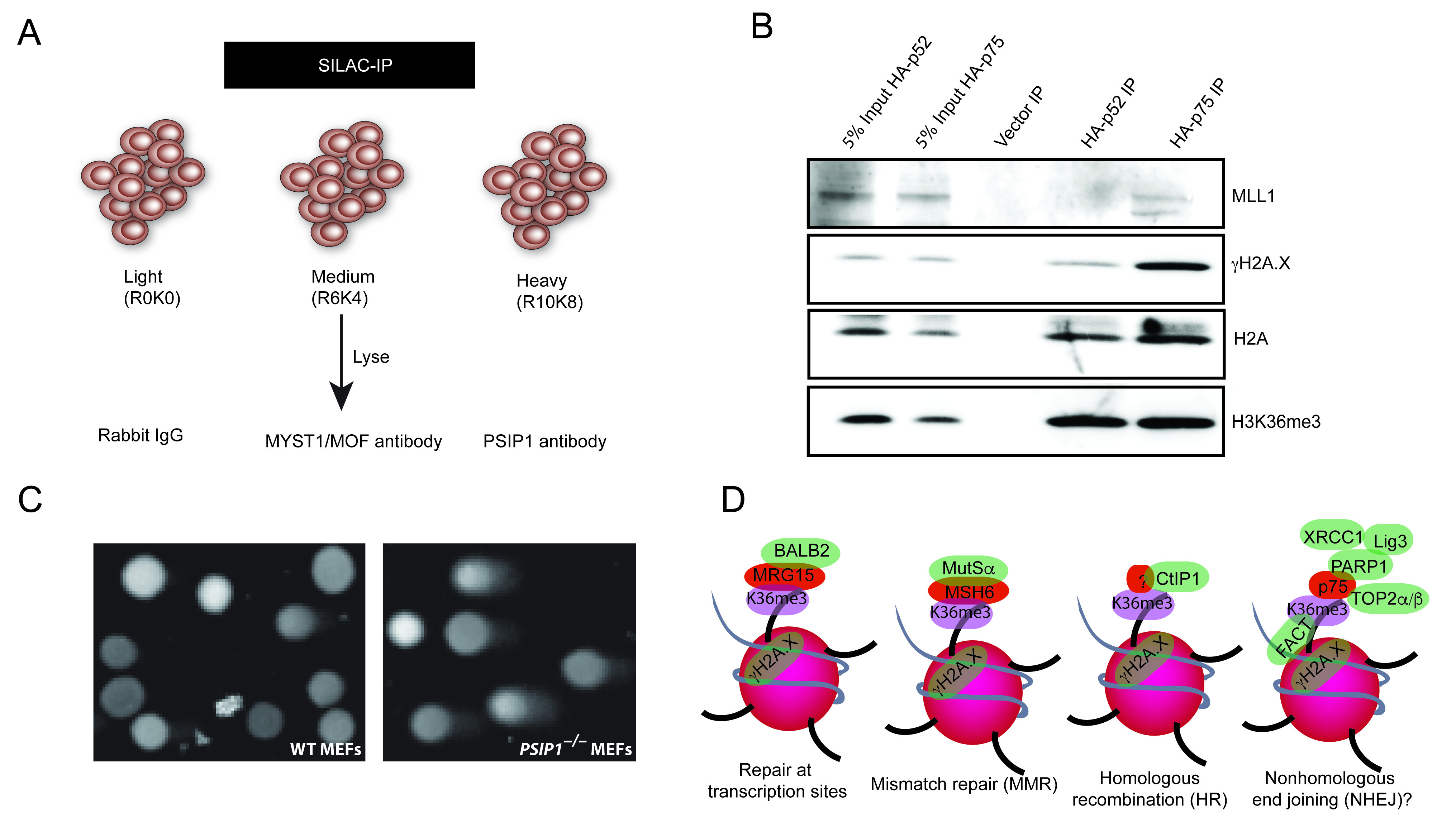
PSIP1 role in facilitating DNA repair. ( **A**) Illustration showing schematics of SILAC immunoprecipitation for using rabbit IgG, anti-MOF and anti-PSIP1 antibodies in cells labelled with light (R0K0), medium (R6K4) and heavy (R10K8) SILAC media. ( **B**) Western blotting with antibodies recognising MLL1, phosphorylated H2A.X (γH2A.X), H2A and H3K36me3, for HA-tag pulldowns from nuclear extracts of *Psip1 ^–/–^* MEFs rescued with HA-p52 and HA-p75. ( **C**) Microscopic images of WT and *Psip1 ^–/–^* MEFs after Comet assay, a representative image from 16 microscopic fields are shown. (n=2 biological replicates). ( **D**) Working model showing various DNA repair proteins that are recruited to H3K36me3 chromatin to modulate repair choice or enhance DNA repair at the site of transcription.

We and others have shown that PSIP1 is a H3K36me3 reader protein, binds to H3K36me3 both *in vitro* and *in vivo* and localises to expressed gene bodies, similar to H3K36me3. The p52 isoform of PSIP1 binds to H3K36me3 and recruits splicing factors to exons of expressed genes ( Pradeepa *et al.*, 2012). Similarly, the p75 isoform binds to H3K36me3 and recruits MLL proteins to expressed HOX genes ( Pradeepa *et al.*, 2014; van Nuland *et al.*, 2013a; van Nuland *et al.*, 2013b). p75 is also shown to promote HDR by recruiting CtIP and RAD51 to DSBs in a H3K36me3 dependent manner ( Aymard *et al.*, 2014; Daugaard *et al.*, 2012; Pfister *et al.*, 2014). In order to comprehensively identify both stable and transient interacting partners of p75, we performed immunoprecipitation (IP) of endogenous p75 protein in cells grown in SILAC media using previously characterised antibodies that specifically pull-down the p75 isoform of PSIP1 ( Pradeepa *et al.*, 2012; Pradeepa *et al.*, 2014). IP with anti-MOF served as an irrelevant control and rabbit immunoglobulin (IgG) served as a negative control. mESCs and MEFs were first labelled for two weeks in light, medium and heavy SILAC cell culture media, followed by IP with negative control (rabbit IgG) anti-MOF (non-relevant chromatin protein) and anti-PSIP1/p75 antibodies, respectively ( Figure 3A). The protein enrichment ratio was then calculated to identify proteins that are quantitatively enriched with p75 and MOF compared to negative control.

### PSIP1/p75 interacts with DNA repair proteins

IP with PSIP1/p75 and MOF antibodies showed PSIP1 and MOF proteins with the highest SILAC ratio over negative control in respective IPs in both MEFs and mESCs ( Table 1; Dataset 2). Proteins identified in PSIP1/p75 IPs are specific to this isoform of PSIP1, as the antibody used for IP is specific to the c-terminal domain of PSIP1/p75, which is absent in the p52 isoform ( Figure 1A). Cell division cycle-associated 7 like (CDC7L), one of the known interacting partners of p75, had the second highest ratio in MEFs ( Hughes *et al.*, 2010). SILAC ratio for γH2A.X was similar to PSIP1 in mESCs, suggesting the co-occurrence of PSIP1 along with γH2A.X at the nucleosomal level. Interestingly, with the exception of XRCC1 all the other DNA repair proteins found in the p75 complex were also associated with H3K36me3 chromatin ( Figure 2). Facilitates active transcription (FACT) heteromeric complex proteins – SSRP1 and SPT16 are associated with H3K36me3, whereas only SPT16 was detected in the PSIP1 complex in this study. However, SSRP1 has been recently shown to interact with PSIP1 ( Lopez *et al.*, 2016), which supports the functional interplay between PSIP1 and the FACT complex in transcriptional elongation and DNA repair. Immunoprecipitation done in mESCs detected fewer proteins and also lower SILAC ratio for p75 ( Table 1; Dataset 2). Intriguingly, other PSIP1 interacting proteins – MLL1, MLL2, Menin, and/or CtIP – were not detected.

**Table 1.** List of proteins that associate with PSIP/p75 in mouse embryonic fibroblasts and stem cells. *proteins also found associated with H3K36me3 chromatin ( Dataset 1); ND, proteins not detected.

Proteins with higher SILAC ratio	Known function	P75/IgG (MEFs)	P75/IgG (mESCs)
PC4 and SFRS1-interacting protein ^*^	Transcription, alternative splicing Promotes DNA repair	93.5	22
Cell division cycle-associated 7-like	Ser/Thr kinase protein	17.7	6
Histone H2A.X ^*^	DNA damage response	ND	23
Isoform Alpha of DNA ligase 3	NHEJ, BER, SSBR	10.9	ND
PARP1 ^*^	DNA repair	8.7	4
XRCC1	single-strand DNA breaks repair	7.3	ND
FACT complex subunit SPT16 ^*^	Nucleosome exchange, DNA repair	5.9	6.3
DNA topoisomerase 1 ^*^	DNA replication & repair	4.6	ND
Top2 alpha ^*^	DNA replication & transcription	9.2	ND
Top2 beta ^*^	DNA replication & transcription	ND	2.9
Tyrosine-protein kinase BAZ1B ^*^	H2A.X kinase	3.6	ND

To validate the interaction of p75 with S139 phosphorylated histone H2AX (γH2A.X), MLL1 and H3K36me3, we performed IP with αHA-tag antibodies in *Psip1* knockout MEFs ( *Psip1 ^–/–^*), which are stably transduced with *HA-Psip1/p75* and *HA-Psip1/p52* ( Figure 3B) ( Pradeepa *et al.*, 2014; Shun *et al.*, 2007). Western blotting of HA IPs with anti-H3K36me3 confirmed the interaction of both p52 and p75 with H3K36me3, which is mediated by a PWWP domain common to both PSIP1 isoforms. Interestingly, we found specific association of a DNA damage marker γH2A.X, with PSIP/p75, but not the p52 isoform. This is consistent with the previously known function of p75 in DNA damage response ( Aymard *et al.*, 2014; Daugaard *et al.*, 2012). PSIP1/p75 is known to interact with MLL1, but it was not detected in our endogenous p75 IP-MS analysis. However, western blotting with anti-MLL1 shows specific interaction of HA-p75, but not HA-p52 with MLL1 ( Figure 3B). These results confirm that although both PSIP1 isoforms are localised to H3K36me3 chromatin, only p75 associates with MLL1, γH2A.X and other DNA repair proteins - consistent with previous reports showing both isoforms of PSIP1 have different protein partners and cellular function ( Daugaard *et al.*, 2012; Ge *et al.*, 1998; Pradeepa *et al.*, 2012; Pradeepa *et al.*, 2014; Pradeepa *et al.*, 2017).

### Higher DNA damage in PSIP1 knockout cells

To examine whether PSIP1 has a direct role in the DNA-damage response, we performed comet assay with WT and *Psip1 ^–/–^* MEFs, which is a sensitive method to measure DNA damage in individual cells ( Olive & Banáth, 2006). Visual scoring of comets showed a significantly higher number of comets with a higher concentration of DNA in *Psip1 ^–/–^* MEFs compared to WT ( Figure 3C). This data supports our previous observation that human cells depleted of p75 show higher levels of unrepaired DNA compared to control, confirming the higher level of unrepaired DNA in cells lacking PSIP1. This strengthens the evidence for a role for PSIP1/p75 in maintaining genomic integrity.

Acetylation of histone H4 at lysine 16 (H4K16ac) and enzymes responsible for most of this modification TIP60 (KAT5) and MOF (KAT8) have been implicated with DNA repair ( Kumar *et al.*, 2013; Sharma *et al.*, 2010; Tang *et al.*, 2013). Recent work suggests a link between H4K16ac and H3K36me3 in DNA damage response ( Li & Wang, 2017). We planned to perform SILAC IP for three different antibodies and were thus able to include anti-MOF IP along with p75 IP and IgG, allowing us to identify the protein partners of MOF along with PSIP1/p75 in mESCs and MEFs. MOF IP also acted as an unrelated chromatin protein control that is known to associate with active genes at regulatory elements ( Li *et al.*, 2012; Taylor *et al.*, 2013). MOF protein has been shown to be associated with both male-specific lethal (MSL) and non-specific lethal (NSL) complexes ( Cai *et al.*, 2010; Li *et al.*, 2009; Mendjan *et al.*, 2006). Canonical MSL proteins were detected in MOF complex and there was no overlap between PSIP1 and MOF complex proteins, which shows the specificity of the IP. Intriguingly, we did not find association of MOF with NSL or DNA repair proteins ( Dataset 2).

Although there have been efforts to study protein partners of PSIP1 and MOF, to our knowledge, this is the first study exploiting the utility of SILAC proteomics to investigate the cellular interactome of these two proteins without using overexpression or epitope tagging. The SILAC immunoprecipitation strategy used here is a very sensitive and powerful means of detecting transient and stable protein partners of chromatin associated proteins.

## Discussion

Purified protein domains that recognise and bind to H3K36me3 can be studied using H3K36 trimethylated histone tail peptides ( Pradeepa *et al.*, 2012). In addition, immunoprecipitation of nuclear extracts with specific peptides followed by SILAC MS has been successfully used to identify methylated histone peptides, including H3K36me3 ( Vermeulen *et al.*, 2010). We now used x-ChIP-qMS, a useful method to identify proteins that interact transiently to histone modifications or chromatin proteins *in vivo*. A similar method was successfully used in *Drosophila* cells to identify MSL associated chromatin proteins and histone modifications ( Wang *et al.*, 2013). In this study, we identified proteins associated with H3K36me3 using x-ChIP-qMS. Further studies upon targeted mutations to histone modifying proteins (writers) or domains that recognises these histone modifications (readers) using gene editing methods will aid in validating the specific interaction of histone modifications and their reader proteins.

SETD2 is the only enzyme responsible for majority of H3K36 trimethylation in mammals, and its depletion reduces H3K36me3 levels, which results in a lower density of FACT subunits SPT16 and SSRP1 ( Carvalho *et al.*, 2013). Our data shows that FACT subunits are associated with both H3K36me3 and p75, suggesting the role of p75 in recruiting the FACT complex to transcribing gene bodies. Indeed, we have previously demonstrated the role of p75 in binding to H3K36me3 at expressed HOX genes and restraining their expression; we propose this regulation occurs at the stage of transcriptional elongation ( Pradeepa *et al.*, 2014). PSIP1/p75 and the SSRP1 complex were recently suggested to be important for the life cycle of HIV ( Lopez *et al.*, 2016). The FACT complex has been shown to facilitate the exchange between H2A.X and H2A ( Heo *et al.*, 2008).

Several pieces of evidence have emerged in recent years for the role of histone modifications in DNA repair. Mammalian Setd2 (homolog of Set2) catalyses H3K36me3 at expressed gene bodies in a transcription dependent manner. H3K36me3 in turn recruits PSIP1 ( Daugaard *et al.*, 2012; Pradeepa *et al.*, 2012; Pradeepa *et al.*, 2014), MRG15 ( Bleuyard *et al.*, 2017; Luco *et al.*, 2010), BS69 ( Guo *et al.*, 2014), DNMT3a ( Dhayalan *et al.*, 2010) and MSH6 ( Li *et al.*, 2013), which modulate transcription, DNA methylation, alternative splicing, and DNA repair choice. A clear association of H3K36me3 with PSIP1 at expressed gene bodies and their association with several DNA repair proteins (this work) and HDR ( Aymard *et al.*, 2014; Daugaard *et al.*, 2012), suggests H3K36me3 and PSIP1 pathway in genome stability.

The constitutive chromatin association of repair factors with H3K36 trimethylated histones that are located at transcribed gene bodies suggests they have a key role in the maintenance of genome stability at the site of transcription. Identification of several DNA repair proteins that interact with PSIP1/p75 that are also associated with H3K36me3 suggests that H3K36me3 and PSIP1 have a wider role in DNA repair pathways. We propose a wide spectrum of roles for SETD2 dependent H3K36me3 and its readers in DNA repair and genome stability than previously suggested ( Figure 3D). PSIP1/p75 is a stress survival protein, also implicated in various cancers including breast, ovarian, prostate and leukaemia, and has also been shown to promote resistance to chemotherapy induced cell death in prostate cancer. Further detailed studies are needed for better understanding of the importance of PSIP1 promoting DNA repair in stress response and in cancers.

## Methods

### Cell lines

Psip1 ^–/–^ and its corresponding WT MEFs ( Pradeepa *et al.*, 2014; Shun *et al.*, 2007) were a kind gift of Prof. Alan Engelman (Dana-Farber Cancer Institute, USA), and were cultured for two weeks in SILAC DMEM media (Dundee Cell Products), containing 10% fetal bovine serum (HyClone, GE Healthcare) and 1% Pen/Strep (Sigma Aldrich). mESCs (OS25, IGMM biostore) were adapted to grow in DMEM media before they were cultured in SILAC DMEM media. MEFs and mESCs to be used as control (rabbit IgG) in the pulldown were grown under R0K0 media; cells used for MOF IP were cultured in R6K4 media; and cells used for P75 IP were cultured in R10K8 media.

### ChIP mass-spectrometry

mESCs were cultured in GMEM as described previously ( Pradeepa *et al.*, 2016). Cells were harvested by trypsinization and fixed immediately with 1% formaldehyde (Thermo Fisher, Cat. 28906) (25°C, 10 min) in PBS, and stopped with 0.125M Glycine. Cross linked cells were re-suspended in Farnham lysis buffer (5 mM PIPES pH 8.0, 85 mM KCl, 0.5% NP-40, Complete Mini EDTA-free protease inhibitor; Roche) for 30 minutes and centrifuged at 228 *g* for 5 minutes at 4°C. Nuclei were resuspended in RIPA buffer (1X PBS, 1% NP-40, 0.5% sodium deoxycholate, 0.1% SDS (filtered 0.2 -0.45 micron filter unit) + Complete Mini EDTA-free protease inhibitor; Roche) and sonicated using a Bioruptor ^®^ Plus sonication device (Diagenode) at full power for 50 minutes (30 seconds on, 30 seconds off) to produce fragments of 200–500 bp. 10 µg of each antibody was incubated with Protein A Dynabeads (ThermoFisher Scientific, 10001D) in 5 mg/ml bovine serum albumin (BSA) in PBS on a rotating platform at 4°C for two hours. An arbitrary concentration of 200 µg chromatin was incubated with antibody bound Dynabeads on a rotating platform at 4°C for 16 hours. Beads were washed 5 times (5 minutes each) on a rotating platform with cold LiCl wash buffer (100 mM Tris pH 7.5, 500 mM LiCl, 1% NP-40, 1% Sodium deoxycholate) and one time with RT TE buffer.

Antibodies used: 5 μg of rabbit IgG (Santa Cruz sc-2025), Histone H3 (rabbit polyclonal, Abcam, Ab 1791), H3K36me3 (rabbit polyclonal, Abcam, Ab 9050) were used per IP. For analysis by mass spectrometry, beads were washed 3 times with Tris-saline buffer, and excess buffer removed. ChIPed complexes were digested on beads, desalted and analysed on a Q-Exactive plus mass spectrometer, as previously described ( Turriziani *et al.*, 2014). Proteins were identified and quantified by MaxLFQ ^17^ by searching with the MaxQuant version 1.5 against the mouse proteome data base (Uniprot). Modifications included C Carbamlylation (fixed) and M oxidation (variable). Bioinformatic analysis was performed with the Perseus software suite.

### Immunoprecipitation

10×14-cm dishes of cells were trypsinized and pelleted, resuspended in 5 ml of ice-cold swelling buffer (10 mM Hepes, pH 7.9, 1.5 mM MgCl 2, 10 mM KCl, 0.5 mM DTT and protease inhibitors (Complete, Roche) for 5 min, and cells were broken open to release nuclei using a pre-chilled Dounce homogenizer (20 strokes with a tight pestle). Dounced cells were centrifuged at 228 *g* (2,000 rpm) for 5 min at 4°C to pellet nuclei and other fragments. The supernatant was discarded. The resulting nuclear pellet was resuspended in 5 ml of RIPA buffer containing 50 mM Tris, pH 7.5, 150 mM NaCl, 1% NP-40, 0.5% Sodium deoxycholate, and protease inhibitors + Benzonase (Novagen, 10 μl/ml), incubated for 30 min on ice, and sonicated briefly on ice (10 × 30 s at full power in bioruptor). Extracts were cleared by centrifugation at 13000 RPM for 10 min at 4°C. Nuclear protein concentrations were measured using a Bradford assay.

Protein A Dynabeads (Life Technologies) were incubated with 5 μg of rabbit IgG (Santa Cruz sc-2025), anti-PSIP/p75 (rabbit polyclonal, Bethyl Laboratories, A300-848A) and anti-MOF (rabbit polyclonal, Bethyl Laboratories A300-992A) in 5% BSA in phosphate buffer saline for two hours, equivalent total protein amounts of extracts were incubated separately with antibodies bound to beads in a rotating platform at 4°C for 30 min. Beads were washed once with RIPA buffer and combined carefully after first wash step. After a further 4 washes, bound proteins were eluted in 4X SDS loading buffer (Life Technologies) with freshly added DTT at 95°C for 5 min. Samples were centrifuged at 11,000 RPM speed for 1 min and supernatant was collect in low binding tube. LC-MS/MS and quantification were carried out by Dundee Cell Products. Briefly, for SDS-PAGE, gel slices per fraction were cut and digested in-gel with trypsin. The purified peptides were then separated (Ultimate U3000, trap-enriched nanoflow LC-system, Dionex), and identified (LTQ Orbitrap XL, Thermoscientific, via nano ES ion source, Proxeon Biosystems). Quantification (MaxQuant, based on 2D centroid of isotope clusters within each SILAC pair) can distinguish between the samples, to give a ratio of protein of interest to IgG. Background proteins would be expected to have a ratio of 1:1 and are therefore disregarded.

### HA-pulldown of p52 and p75

PSIP1 knockout MEFs stably rescued with HA-PSIP1/p52 and HA-PSIP1/p75 were immunoprecipitated with anti-HA antibodies. 5% input lysate and IPed proteins were separated on Novex 4–20% gels and transferred to PVDF membranes. Western blotting was performed by immunoblotting with MLL1 (Active Motif, 61295), gH2A.X (Millipore, 05-636), H2A and H3K36me3 (Abcam, ab9050) antibodies.

### Comet assay

Comet assays for WT and *Psip1* knockout MEFs were performed using Comet Assay kit (OxiSelect™), according to the manufacturer’s instructions.

## Data availability

Raw data for this study are available from OSF http://doi.org/10.17605/OSF.IO/UAX7G ( Pradeepa, 2017). Dataset 1: SILAC results for MEFs; Dataset 2: SILAC results for OS25 cells, WT MEFs Comet assay data, PSIP1 KO Comet assay data, and uncropped blots for Figure 3B.
