# Supplementary material for: H3K36me3 and PSIP1/LEDGF associate with several DNA repair proteins, suggesting their role in efficient DNA repair at actively transcribing loci
Source: Wellcome Open Res. 2021 Sep 14;2:83. Originally published 2017 Sep 12. [Version 4] doi: 10.12688/wellcomeopenres.11589.4 (PMC8422350; doi:10.12688/wellcomeopenres.11589.4)
Supplement: Peer review history

# Peer review: Reviewer response for version 3

**DOI:** 10.21956/wellcomeopenres.18677.r45684

**Published:** 2021-09-06

**Authors:** Zeger Debyser

**Affiliations:** 1 Molecular Virology and Gene Therapy, Department of Pharmacological and Pharmaceutical Sciences, KU Leuven, Leuven, Belgium

In this revised version, the authors address questions asked by referees.

 I still have 2 comments:

1. They should explain the results of the comet assay in text or legend for people not familiar with this assay.
2. Also in line with previous comments. Although the data shown suggest an interaction of H3K36me3 and LEDGF/p75 with DNA repair proteins, the analysis used does not discriminate between direct interaction and indirect interaction; e.g. binding partners of LEDGF/p75 may interact with repair proteins. So the claims that LEDGF/p75 by itself is involved in DNA repair should be played down a bit. Therefore a direct interaction with recombinant and purified proteins ought to be shown.

Is the work clearly and accurately presented and does it cite the current literature?

Yes

If applicable, is the statistical analysis and its interpretation appropriate?

Yes

Are all the source data underlying the results available to ensure full reproducibility?

Yes

Is the study design appropriate and is the work technically sound?

Yes

Are the conclusions drawn adequately supported by the results?

Partly

Are sufficient details of methods and analysis provided to allow replication by others?

Yes

Reviewer Expertise:

Molecular virology, epigenetics, LEDGF/p75

I confirm that I have read this submission and believe that I have an appropriate level of expertise to confirm that it is of an acceptable scientific standard.

# Peer review: Reviewer response for version 2

**DOI:** 10.21956/wellcomeopenres.14001.r26978

**Published:** 2017-10-23

**Authors:** Jan De Rijck

**Affiliations:** 1 Department of Pharmaceutical and Pharmacological Sciences, KU Leuven, Leuven, Belgium

Since mass spec data are generally known to contain a lot of false positive results, both reviewers requested that the most important mass spec hits should be independently confirmed, by for instance, IP experiments. From all experiments requested by the reviewers, this is the absolute minimum that should be added to the manuscript before approval.

Is the work clearly and accurately presented and does it cite the current literature?

Partly

If applicable, is the statistical analysis and its interpretation appropriate?

Yes

Are all the source data underlying the results available to ensure full reproducibility?

Partly

Is the study design appropriate and is the work technically sound?

Partly

Are the conclusions drawn adequately supported by the results?

Partly

Are sufficient details of methods and analysis provided to allow replication by others?

Yes

Reviewer Expertise:

NA

I confirm that I have read this submission and believe that I have an appropriate level of expertise to confirm that it is of an acceptable scientific standard, however I have significant reservations, as outlined above.

# Response

**Published:** 2021-05-17

**Authors:** Pradeepa Pradeepa

**Affiliations:** Queen Mary, University of London, UK

As we agreed earlier we now have revised the manuscript with new data that supports the interaction of PSIP1 with PARP1 which is one of the key proteins involved in DNA repair. 

 New data:

 We performed immunoprecipitation with PSIP/p75 specific antibody, western blotting of PSIP/p75 IPed complex showed specific enrichment of PARP1 and hnRNPU/SAFA in p75 IP but not SSRP1 one of the subunit of FACT. We also tested several antibodies, unfortunately, many antibodies did not work in our hands. We now believe we have sufficient evidence that supports a wider role of H3K36me3 and PSIP1/p75 in DNA repair pathways

# Peer review: Reviewer response for version 2

**DOI:** 10.21956/wellcomeopenres.14001.r26979

**Published:** 2017-10-13

**Authors:** Deepak Jha

**Affiliations:** 1 Division of Pediatric Hematology and Oncology, Boston Children's Hospital and Dana-Farber Cancer Institute, Boston, MA, USA

I do not have any further comments.

Is the work clearly and accurately presented and does it cite the current literature?

Partly

If applicable, is the statistical analysis and its interpretation appropriate?

Yes

Are all the source data underlying the results available to ensure full reproducibility?

Yes

Is the study design appropriate and is the work technically sound?

Yes

Are the conclusions drawn adequately supported by the results?

Partly

Are sufficient details of methods and analysis provided to allow replication by others?

Yes

Reviewer Expertise:

Epigenetics, Genome Stability, Cell Cycle, Cancer Epigenetics, Hematopoiesis

I confirm that I have read this submission and believe that I have an appropriate level of expertise to confirm that it is of an acceptable scientific standard.

# Peer review: Reviewer response for version 1

**DOI:** 10.21956/wellcomeopenres.12519.r25938

**Published:** 2017-09-25

**Authors:** Deepak Jha

**Affiliations:** 1 Division of Pediatric Hematology and Oncology, Boston Children's Hospital and Dana-Farber Cancer Institute, Boston, MA, USA

The overarching goal of this manuscript by Pradeepa et.al is to identify if PSIP1 isoforms provide a binding platform for DNA repair proteins, which would explain how H3K36me3 functions in DNA repair. They use proteomics to identify, in a somewhat unbiased manner, the interacting partners of H3K36me3 chromatin, and PSIP1/p75. They found a substantial overlap between the binding partners of H3K36me3 and p75. Furthermore, they perform co-IP with Y-H2A.X to validate their Ip-mass spectrometry data. Finally, they show that *Psip1*-/- cells have higher level of unrepaired DNA damage, thereby alluding to a role for *Psip1* -/- in DNA repair.

 Specific Comments:

1. As far as I can tell, all of the mass spectrometry experiments have been performed without the induction of any DNA damage. This implies that one is looking at some combination of intrinsic DNA damage associated binding partners, which would be heavily influenced by transcriptional biases (related to gene length, transcriptional frequency and exons), and cell cycle phases. To directly make a conclusive statement regarding if *PSIP1 *acts as a scaffold for recruiting DNA repair proteins, it would be beneficial to monitor the interaction(s) with and without DNA damage. Given the extensive rewiring of cellular signaling after DNA damage, having a more direct readout of differential interacting partners would be more beneficial to refine the various models presented in figure 3d.
2. Figure 3 can be improved by testing some other key interacting partners from table 1. Specifically, components of FACT complex and PARP1 should be tested in co-IP experiments since both of them are key regulators of DNA repair as well.
3. Fig. 3: Additional evidence for unrepaired DNA damage/abrogated DNA damage signaling in *Psip1-/- *should be provided. E.g. Y-H2A.X retention kinetics after DNA damage in *Psip1-/- *cells.
4. Datasets1 and 2 should be labeled in the same manner as the OSF files.
5. The authors state “Interestingly, with the exception of….” and cite Fig.2 for this. They should also refer to table1, alongside Fig.2 to clarify the overlaps.
6. Can the authors phenocopy fig.3c with a PWWP mutant of p75 in order to directly test if H3K36me3- p75 are part of the same mechanism in DNA repair?

Is the work clearly and accurately presented and does it cite the current literature?

Partly

If applicable, is the statistical analysis and its interpretation appropriate?

Yes

Are all the source data underlying the results available to ensure full reproducibility?

Yes

Is the study design appropriate and is the work technically sound?

Yes

Are the conclusions drawn adequately supported by the results?

Partly

Are sufficient details of methods and analysis provided to allow replication by others?

Yes

Reviewer Expertise:

Epigenetics, Genome Stability, Cell Cycle, Cancer Epigenetics, Hematopoiesis

I confirm that I have read this submission and believe that I have an appropriate level of expertise to confirm that it is of an acceptable scientific standard, however I have significant reservations, as outlined above.

# Peer review: Reviewer response for version 1

**DOI:** 10.21956/wellcomeopenres.12519.r25937

**Published:** 2017-09-20

**Authors:** Jan De Rijck

**Affiliations:** 1 Department of Pharmaceutical and Pharmacological Sciences, KU Leuven, Leuven, Belgium

Several manuscripts have suggested a role for LEDGF/p75 in transcriptional elongation and DNA repair. Since LEDGF/p75 is known to read H3K36me3, a marker of active chromatin present in the body of genes, and since both H3K36me3 and LEDGF/p75 have been linked to DNA repair it is tempting to speculate that both are part of the same molecular mechanism. Pradeepa *et al.* tried to tackle this question by analysing and comparing the proteome of H3K36me3 and LEDGF/p75.

 Comments:

- Recently, LEDGF/p75 has been suggested to interact with H3K36me2 next to H3K36me3 (Zhu *et al* 2016 and Okuda, 2014). Some even claim that this interaction is preferred over H3K36me3. In this regard, LEDGF/p75 can also be found in promotor regions. Although this remains a matter of debate it should be mentioned in the introduction.
- The datasets in the supplemental material are not clear to me. Dataset 1 and 2 are mentioned but not clearly linked to the supplements. I could not find dataset 1.
- Although DNAse is used to overcome DNA bridging in the IP experiments, the xChip experiments are inevitably prone to DNA bridging. One can only claim that these proteins were in the neighbourhood of H3K36me3 marks. This should be clearly indicated in the discussion.
- In the text it is claimed that there is a strong overlay between both datasets in DNA repair proteins. The authors refer to figure 2 to support this statement. However, this figure only mentions H3K36me3 hits. The general overlay between both set could be presented in a better way.
- LEDGF is a major hit in the H3K36me3 xChIP. Looking at the sequenced peptides, can you speculate whether this was p52 or p75?
- Confirmation of some of the important hits by co-immunoprecipitation experiments as presented in figure 3b would strengthen the credibility of the manuscript.
- The fact that LEDGF/p75 has an effect on DNA damage response (figure 3c) is not new. However, neither the present manuscript nor previous manuscripts from other groups could provide a direct link. The authors claim that LEDGF/p75 and not p52 affects DNA repair. As such, there is a good chance that a protein binding to the IBD domain is responsible for this effect. Proteins binding the IBD are characterised by an IBM motif (Tesina *et al*, 2015).
   
  - Could the authors rescue the DNA repair defect in -/- cells by a mutant LEDGF/p75 protein not able to interact with IBM proteins?
  - CDC7/ASK is the only protein in table 1 with an IBM motif. Can HA-LEDGF/p75 rescue DNA repair upon knockdown ofCDC7/ASK?

These data could provide a direct link between LEDGF/p75 and DNA repair.

Is the work clearly and accurately presented and does it cite the current literature?

Partly

If applicable, is the statistical analysis and its interpretation appropriate?

Yes

Are all the source data underlying the results available to ensure full reproducibility?

Partly

Is the study design appropriate and is the work technically sound?

Partly

Are the conclusions drawn adequately supported by the results?

Partly

Are sufficient details of methods and analysis provided to allow replication by others?

Yes

Reviewer Expertise:

NA

I confirm that I have read this submission and believe that I have an appropriate level of expertise to confirm that it is of an acceptable scientific standard, however I have significant reservations, as outlined above.

# Response

**Published:** 2017-10-11

**Authors:** Pradeepa Pradeepa

**Affiliations:** Queen Mary, University of London, UK

We thank Dr. Jan De Rijck for critically reviewing this paper and also suggestions to improve the credibility of the manuscript. Please see our responses below for each of the specific points.

 Recently, LEDGF/p75 has been suggested to interact with H3K36me2 next to H3K36me3 (Zhu *et al* 2016 and Okuda, 2014). Some even claim that this interaction is preferred over H3K36me3. In this regard, LEDGF/p75 can also be found in promoter regions. Although this remains a matter of debate it should be mentioned in the introduction. *We have mentioned this is the introduction*


*  *

- The datasets in the supplemental material are not clear to me. Dataset 1 and 2 are mentioned but not clearly linked to the supplements. I could not find dataset 1.

*We have added the datasets now clearly labeled*


*  *

- Although DNAse is used to overcome DNA bridging in the IP experiments, the xChip experiments are inevitably prone to DNA bridging. One can only claim that these proteins were in the neighborhood of H3K36me3 marks. This should be clearly indicated in the discussion.

*We completely agree with the reviewer’s point, hence say these proteins are associated with H3K36me3. *

- In the text, it is claimed that there is a strong overlay between both datasets in DNA repair proteins. The authors refer to figure 2 to support this statement. However, this figure only mentions H3K36me3 hits. The general overlay between both sets could be presented in a better way.

* We have now referred to Table 1 and Figure 2 and dataset 1 *

- LEDGF is a major hit in the H3K36me3 xChIP. Looking at the sequenced peptides, can you speculate whether this was p52 or p75?

*We have now looked into this and found p75 peptides in the mass spec data. So we cannot rule out the presence of shorter p52 isoform along with the p75 isoform. We didn't find a short peptide that distinguishes between two isoforms. So our data shows both isoforms are likely to present in the H3K36me3 domain. *

- Confirmation of some of the important hits by co-immunoprecipitation experiments as presented in figure 3b would strengthen the credibility of the manuscript.

*We completely agree with the suggested confirmatory experiments by both reviewers will strengthen the credibility of the manuscript. We will be performing these experiments when we have resources, and we will update the manuscript.   *

- The fact that LEDGF/p75 has an effect on DNA damage response (figure 3c) is not new. However, neither the present manuscript nor previous manuscripts from other groups could provide a direct link. The authors claim that LEDGF/p75 and not p52 affects DNA repair. As such, there is a good chance that a protein binding to the IBD domain is responsible for this effect. Proteins binding the IBD are characterized by an IBM motif (Tesina *et al*, 2015).

*It is an interesting point; we have now discussed this in the manuscript *

- Could the authors rescue the DNA repair defect in -/- cells by a mutant LEDGF/p75 protein not able to interact with IBM proteins?

*Thanks for suggesting this experiment, we would love to do this experiment in the future, but not for this manuscript  *

- CDC7/ASK is the only protein in table 1 with an IBM motif. Can HA-LEDGF/p75 rescue DNA repair upon knockdown ofCDC7/ASK?

These data could provide a direct link between LEDGF/p75 and DNA repair.

*We appreciate the suggestions to improve the manuscript by both the reviewers. Due to limited resources, we cannot perform the suggested experiments at this time. However, we will perform the suggested experiments in future. We wished to share our data with this publication to wider readers at the earliest time point.*
